# Cancer care during the COVID-19 pandemic: a perspective from Saudi Arabia

**DOI:** 10.3332/ecancer.2020.1076

**Published:** 2020-07-17

**Authors:** Saleh A Alessy, Elizabeth A Davies, Abdul-Rahman Jazieh

**Affiliations:** 1 Cancer Epidemiology, Population and Global Health, Comprehensive Cancer Centre, Faculty of Life Sciences & Medicine, King's College London, UK; 2 Public Health Department, College of Health Sciences, Saudi Electronic University, Riyadh, 11426, Saudi Arabia; 3 Department of Oncology, King Saud bin Abdulaziz University for Health Sciences, Riyadh, 11426, Saudi Arabia; 4 King Abdullah International Medical Research Center, Ministry of National Guard Health Affairs, Riyadh, 11426, Saudi Arabia; a https://orcid.org/0000-0003-4588-7410

**Keywords:** cancer, care, COVID-19, Saudi Arabia

## Abstract

The coronavirus disease 2019 (COVID-19) pandemic continues to disrupt many healthcare settings worldwide including cancer care. COVID-19 has been associated with worse outcomes amongst cancer patients. Saudi Arabia has experienced several Middle East respiratory syndrome coronavirus (MERS-CoV) outbreaks that affected the continuity of cancer care. In this paper, we describe how Saudi Arabia responded to COVID-19, how cancer care was re-restructured during this pandemic and how the recent MERS-CoV experience may have improved the Saudi response to COVID-19.

## Introduction

The COVID-19 pandemic continues to escalate, posing a worldwide public health threat across many healthcare systems [1]. COVID-19 has caused significant disruption to many secondary healthcare services, including cancer care [2]. Interruptions to cancer care can be expected to have a long-term impact on cancer patients’ diagnoses, their care management and subsequent outcomes [3, 4]. In addition, cancer patients are usually susceptible to infectious diseases, which can be life-threatening for this population. Vulnerability is due to the severity of disease, together with underlying co-morbidities and cancer treatment side effects [5]. Healthcare systems around the world have taken different approaches to control COVID-19 [6] and to ensure the continuity of other urgent health care such as cancer care [2]. Plans have varied based on several factors, including the capacity of each healthcare system, experiences with the outbreaks of previous infectious diseases and specific economic, political and social factors [6].

## Background

Saudi Arabia is a large Middle Eastern country, which extends over four-fifths of the Arabian Peninsula. It has a relatively young population with only around 5% aged over 65 [7]. The last estimated population size for Saudi Arabia was around 34 million in 2020 [7]. This number includes 21 million Saudi nationals (51% males and 49% females) and 13 million non-Saudi nationals (69% males and 31% females) [7]. Although the healthcare system in Saudi Arabia consists of public, private and non-profit sectors, the Saudi government announced a new policy of free COVID-19 treatment for all at the beginning of the pandemic [8]. In addition, a high-level committee consisting of 24 government agencies, headed by the Minister of Health, was appointed to develop the pandemic control plan [9]. The plan involved preparing 34 hospitals, with around 8,000 intensive care unit (ICU) beds, 2,000 isolating rooms and 20 national and regional laboratories across the kingdom [9]. The Ministry of Health also used its pre-existing online application ‘Seha’ to provide virtual medical consultations and, in collaboration with other government agencies, launched a new tracking and tracing app ‘Tataman’ [9].

## Preparedness

In terms of social distancing, the government of Saudi Arabia has taken several serious steps to mitigate COVID-19 beginning by cancelling all large events and closing schools early in March [9]. Other measures included halting all national and international flights and isolating all incoming citizens who returned from abroad for 14 days. Saudi Arabia is a Muslim country and a home for holy sites that are visited by millions of Muslims every year. Umrah (an Islamic ritual performed by thousands of Muslims daily in the city of Makkah) was suspended for this year. In addition, five prayers performed daily in thousands of mosques across the kingdom were also suspended between late March and 1 June 2020 [8]. In March, the Saudi government gradually imposed lockdowns and curfews with financial penalties on lawbreakers [8]. These measures have now been gradually relaxed, beginning from 1 June 2020 with a new approach beginning to mass testing, tracing and isolating [9]. The Ministry of Health has issued many guidelines and messages to the public across all social media platforms alongside a daily press conference to raise awareness about the important measures needed to control COVID-19. The Saudi Center for Disease Control and Prevention (SCDC) continues to play a key role in issuing COVID-19 prevention measures and treatment protocols based on research drawn from the international literature [10].

## Epidemiology of COVID-19 in Saudi Arabia

**Figure 1.**
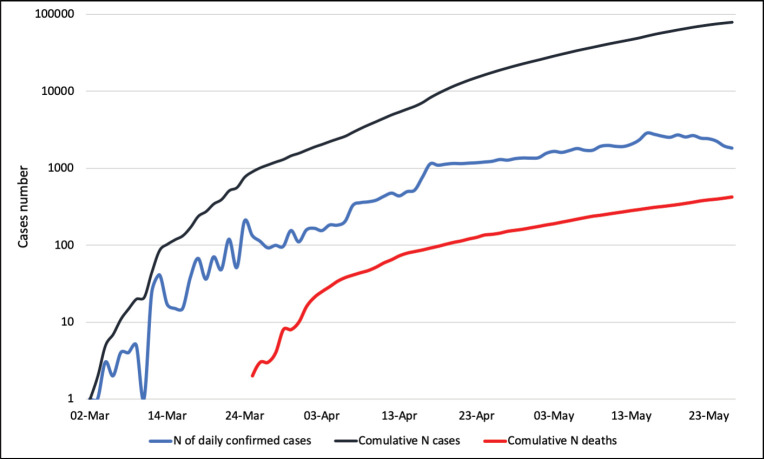
COVID-19 statistics for Saudi Arabia. These data are based on the daily cases announced by the Ministry of Health (last updated on the 1 June 2020). Data source: [11], last accessed on the 2 June 2020; Abbreviation: N = number.

**Figure 2.**
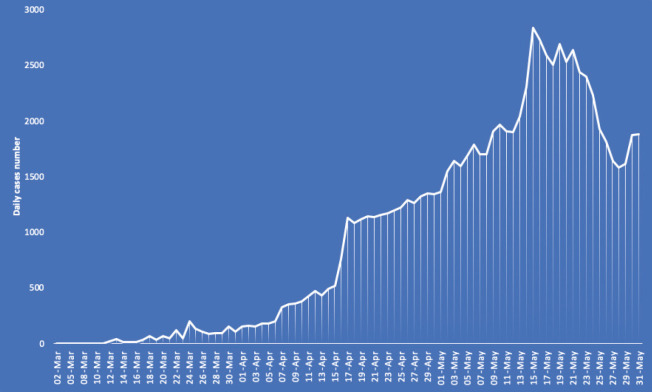
Daily reported COVID-19 cases in Saudi Arabia. These data are based on the daily cases announced by the Ministry of Health (last updated on the 1 June 2020). Data source: [11], last accessed on 2 June 2020.

The first COVID-19 case was confirmed in Saudi Arabia on 2 March 2020. The total number of reported cases of COVID-19 has reached 87,000 cases with 525 deaths (last updated on 1 June 2020) (Figures 1 and 2). Whilst there are still no data available on COVID-19 cases by their age group and other demographic factors in Saudi Arabia, the lower mortality rate might be a reflection of the young population in the kingdom.

## Previous experience with outbreaks

Before this pandemic, Saudi Arabia had recent experience of controlling an infectious disease epidemic that had consequences for many healthcare settings including cancer care [12]. The Middle East respiratory syndrome coronavirus (MERS-CoV) was first discovered in 2012 in Saudi Arabia and caused several human-to-human outbreaks in hospitals in Jeddah and Riyadh between 2014 and 2015 [13]. Although MERS-CoV cases continue to be registered sporadically in Saudi Arabia, the initial epidemic had the effect of preparing public health agencies in Saudi Arabia for similar situations in the future. The previous MERS-CoV endemic, therefore, led to several public health agencies being established including 1) the Command and Control Centre (CCC), 2) the SCDC and 3) the National Health Laboratory (NHL) [8]. In addition, the Saudi experience with MERS-CoV led to the implementation of better infection control measures and better coordination of care for vulnerable patients such as those with cancer [5, 12].

## Cancer care continuity

As in many countries, cancer care in Saudi Arabia has been interrupted by the COVID-19 pandemic. Cancer is gradually becoming an increasing part of healthcare use in the kingdom. The total annual cancer incidence was 16,859 in 2016, with the age-standardised incidence rate (ASR) of 74.7 per 100,000 in men and 91.3 in women [14]. In addition, the care of cancer survivors accounts for a large part of oncology service use in the Kingdom, with the 5-year prevalence of people who have had cancer being estimated at be around 70,000 patients in 2018 [15]. There are currently 23 governmental centres providing cancer care across Saudi Arabia, and these services are provided free for citizens without any out of pocket costs.

Saudi Arabia has learned many lessons about how to manage cancer care during the life-threatening MERS-CoV outbreak. A total of 2,499 MERS-CoV cases were confirmed from 27 countries since 2012 resulting in 858 deaths (34.3% mortality), with the majority of these deaths (780 deaths) being reported in Saudi Arabia [13]. Mortality for those known to be infected with MERS-CoV was higher in patients older than 65 years and patients with the other comorbidities such as cancer, immunosuppression, chronic lung disease and diabetes [12, 13]. These lessons included the need to ensure the continuity of care, minimise harm from acquiring life-threatening infection and reduce the number of situations, in which treatment became interrupted [12]. In March 2020, the Saudi Arabia National Cancer Institute (SANCI) issued a guideline for caregivers and cancer facilities to minimise the impact of COVID-19 on cancer patients [16]. The guideline, last updated on April, set out several prevention and management steps. This included educating staff in cancer care settings about COVID-19 symptoms, ensuring preventative infection control measures within each setting, providing all staff and cancer patients on treatment with official transportation permits during curfew or lockdown and providing virtual care and services [16, 17].

For cancer care facilities, the infection control measures included limiting hospital access to one entry point, setting up a screening team and establishing triage stations at the entrances of outpatient clinics and radiation therapy units [16]. To reduce the risk of patients acquiring COVID-19, several steps were taken for non-urgent onsite cancer care. Physicians were advised to meet virtually for multidisciplinary team meetings, whereas patients’ routine follow-up visits and non-urgent surgery were postponed or replaced by virtual clinics [16]. Cancer patients’ support services were delivered through a virtual platform, and drive-through or mail delivery was used to deliver cancer patients’ treatments [16].

Moreover, SANCI provided specific instructions on how to manage cancer patients based on the confirmation of having or not having the COVID-19 infection. Patients who were confirmed not to have COVID-19 infection should start or continue the systemic cancer treatment, whereas patients’ attendances at radiotherapy facilities were reduced by adopting hypo-fractionated regimens [16]. However, the management of cancer patients confirmed to have COVID-19 included admission to a designated COVID-19 unit, delaying cancer treatment until complete recovery and assigning a designated team which includes an infectious disease consultant to each confirmed patient care [16].

For example, the National Guard Health Affairs, which operates several oncology centres across the kingdom, launched virtual oncology clinics on March 18 to serve eligible patients who do not need to receive in-person care. All elective cancer surgery, routine follow-up and survivorship visits were postponed. In addition, cancer medications were delivered to patients’ homes. An ongoing study by Jazieh and colleagues compares cancer care delivery and treatment during COVID-19 in 356 centres within 53 countries, including 28 oncology settings in Saudi Arabia. The early analysis (unpublished) revealed the implementation of similar interventions with limited variation in the majority of centres including the Saudi centres. These interventions are consistent with the guidelines published by different oncology entities involving experts from Saudi Arabia [18, 19].

Oncologists in Saudi Arabia and some other Gulf states appeared to show good awareness and use of virtual cancer clinics during the COVID-19 pandemic [20]. However, due to the complexity of cancer care, oncologists still face some challenges such as the lack of physical examination, the co-ordination of MDTs virtually and the lack of patient’s awareness and access to virtual clinics [20]. In addition, cancer screening services provided by governmental and cancer charities are not always trusted by the population as being safe from COVID-19. This is expected to contribute to an increase in the pre-existing issue of late-stage cancer diagnosis in the kingdom [21]. It is also hypothesised that patients might decline to come to care settings for diagnosis, surgery, or chemotherapy due to their fear of acquiring the COVID-19 [22]. Data on the utilisation of these services and mortality amongst cancer patients from COVID-19 are not yet available to begin to judge the actual impact. There is still a need for research to assess the long-term impact of COVID-19 on cancer patients across the entire care continuum. A local research is also needed on the Saudi healthcare system and population to determine whether there are risk factors associated with cancer and COVID-19 on patients’ outcomes such as comorbidities and previous exposure to MERS-COV. Recently, a plan to establish a national registry for COVID-19 in cancer patients is being implemented to facilitate the related COVID-19 cancer research.

## The way forward

Cancer care in Saudi Arabia appeared resilient during the COVID-19 pandemic. Many lessons were learned from MERS-COV which strengthened the public health system and enabled the prior operational experience of managing cancer care during an infectious disease outbreak. Longer term follow-up research is also needed to determine the impact on cancer services utilisation, cancer diagnosis and subsequent survival.

## Conflict of interest

The authors declare that they have no conflicts of interest

## Funding

This work did not receive any funding.
